# Interstitial Lung Disease Phenotypes and Predictive Risk Factors in Primary Sjögren’s Syndrome

**DOI:** 10.3390/jcm13164963

**Published:** 2024-08-22

**Authors:** Gaetano La Rocca, Francesco Ferro, Gianluca Sambataro, Elena Elefante, Giovanni Fulvio, Inmaculada Concepción Navarro, Michele Moretti, Chiara Romei, Marta Mosca, Chiara Baldini

**Affiliations:** 1 Rheumatology Unit, Department of Clinical and Experimental Medicine, University of Pisa, Via Roma 67, 56126 Pisa, Italy; 2 Rheumatology Unit, Department of Clinical and Experimental Medicine, AOE Cannizzaro, University of Catania, Via Messina 829, 95126 Catania, Italy; 3 Artroreuma S.R.L., Rheumatology Outpatient Clinic Associated with the National Health System, Corso S. Vito 53, 95030 Catania, Italy; 4 Radiodiagnostic Unit 2, Department of Diagnostic Imaging, University of Pisa, Via Paradisa 2, 56124 Pisa, Italy

**Keywords:** interstitial lung disease, primary Sjögren’s syndrome, clinical phenotypes, HRCT patterns, risk factors, prognosis

## Abstract

**Background/Objectives:** The prevalence of Interstitial Lung Disease (ILD) and risk factors for its development in patients with primary Sjögren’s syndrome (pSS) are still debated, possibly due to the existence of heterogeneous pSS-related ILD phenotypes. The aims of this study were: 1. To investigate the prevalence and predictive factors for ILD development in a single-center pSS cohort; 2. To characterize different pSS-ILD phenotypes. **Methods:** Clinical, laboratory and imaging data of pSS patients attending our center from January 2019 to September 2023 were retrospectively analyzed. ILD presence was confirmed on HRCT. **Results:** Forty-three out of 474 enrolled pSS patients presented ILD (M:F = 6:37), accounting for an overall ILD prevalence of 9.1%. In 19 cases, ILD was the first manifestation of pSS (ILD-onset), while in 24 ILD was diagnosed after pSS (ILD-incident). Compared to ILD-onset, ILD-incident patients more often presented pSS-related hematologic abnormalities (*p* = 0.012), cutaneous involvement (*p* = 0.027), inflammatory arthralgias (*p* = 0.026), C4 hypocomplementemia (*p* = 0.012) and positive RF (*p* = 0.031). On the other hand, ILD-onset patients were significantly older at pSS diagnosis (*p* = 0.008) and presented more severe fibrosis on HRCT (*p* = 0.008). On the univariate analysis, higher ESSDAI (*p* = 0.011), Raynaud’s phenomenon (*p* = 0.009), anti-Ro52 (*p* = 0.031), hypergammaglobulinemia (*p* = 0.011), Rheumatoid Factor (RF) (*p* = 0.038) and C4 hypocomplementemia (*p* = 0.044) at baseline were associated to ILD development during follow-up. On the multivariate analysis, the ESSDAI at baseline (*p* = 0.05) and Raynaud’s phenomenon (*p* = 0.013) at baseline were the only independent predictors of ILD development. **Conclusions:** ILD is a relatively common and clinically heterogenous pSS manifestation. Elevated disease activity at pSS onset is a risk factor for ILD development, prompting careful follow-up and intriguingly suggesting that immunomodulatory therapies may prevent ILD.

## 1. Introduction

Primary Sjögren’s Syndrome (pSS) is a systemic autoimmune disease that mainly affects exocrine glands resulting in sicca syndrome and has classically been considered a relatively benign condition compared to other connective tissue diseases [1]. However, the presence of extra-glandular manifestation in 20–40% of patients radically changes this paradigm, identifying a distinct subset of patients with potentially severe prognosis [2,3].

Interstitial lung disease (ILD) is among the most serious and common extra-glandular manifestations of pSS [4]. The most common clinical symptoms of ILD, namely chronic cough and exertional dyspnea, may present in pSS patients only after a long subclinical phase [5]. High Resolution Computed Tomography (HRCT) is currently considered the gold standard diagnostic technique for the screening of ILD in pSS patients [6,7]. It also provides valuable information on ILD pattern, extension and severity, therefore playing a key role for the prognostic assessment and management [6,8,9]. Histologic examination is not routinely required in pSS patients, and whether a more precise histologic characterization of ILD pattern improves the prognostic stratification and treatment strategies is still debated. However, lung biopsy is still essential and recommended in case of diagnostic uncertainty on ILD etiology or suspicion of associated infectious or malignant processes [7,10]. Surgical Lung Biopsies have long been considered the preferred approach, though recent studies have suggested transbronchial cryo-biopsy as presenting a more favorable profile in terms of diagnostic accuracy and safety [11,12,13].

In spite of the great impact of ILD in terms of morbidity and mortality burden [14], its actual prevalence in pSS patients is still a matter of debate and varies dramatically across different studies [15], with most recent literature fixing it around 20% [16].

Similarly, results from the largest studies investigating demographic, clinical and serological characteristics associated with ILD development in pSS patients are often conflicting [17]. For instance, serological positivity for anti-SSA, typically linked to most pSS extra=glandular manifestations, has been associated with ILD by some studies [18], but not others [19,20,21]. In other works, assessing separately anti-Ro60 and anti-Ro52 autoantibodies, the latter were associated to ILD presence, as is the case for other connective tissue diseases [22,23].

Numerous reasons may account for the aforementioned discrepancies on prevalence and risk factors for ILD in the previous works, including the lack of uniformity in ILD definition and screening strategies, and the use of different sets of criteria for pSS classification. However, another important confounding factor is that pSS-ILD probably encompasses heterogenous clinical and radiological phenotypes and a variable relative prevalence of these phenotypes in the different cohorts may have affected research findings.

In this regard, it is common knowledge that in almost 50% of cases ILD precedes or occurs concomitantly with pSS diagnosis (ILD-onset). We have recently reported that ILD-onset pSS patients are older, exhibit milder sicca symptoms and less structural abnormalities of salivary glands on ultrasound compared to classical sicca-onset pSS patients [24]. In contrast, ILD developing after pSS diagnosis (ILD-incident) has been less extensively characterized.

The aims of the present work were therefore to investigate the prevalence of ILD in our pSS cohort and to characterize the different pSS-ILD phenotypes comparing patients developing ILD during disease course with those of patients diagnosed with ILD before or concurrently with pSS. Finally, we explored baseline risk factors for ILD development during the pSS course.

## 2. Materials and Methods

### 2.1. Patients

This was a single-center retrospective cohort study, including pSS patients in follow-up from January 2019 to September 2023 in the Rheumatology Department of the University Hospital of Pisa (AOUP), Italy. We only enrolled consecutive pSS patients who had been screened for ILD presence by clinical examination plus at least one among chest X-ray and/or Pulmonary Function Tests (PFT) and/or chest High Resolution Computed Tomography (HRCT). Of note, since the publication of the 2021 Consensus Guidelines for Evaluation and Management of Pulmonary Disease in Sjögren’s [7], all pSS patients in our center underwent chest X-ray and PFT at diagnosis, and HRCT was performed in all cases if lung involvement was suspected. All patients included in the study fulfilled the ACR/EULAR 2016 criteria for the classification of pSS [25] and cases of pSS overlapping with another connective tissue disease (CTD) were excluded. Patients with a diagnosis of smoking related pulmonary disease were also excluded. Moreover, only pSS patients with available autoantibody testing by immunoblot-based panels for connective tissue diseases were included and complete results were recorded.

For all patients we collected data regarding demographics, clinical and laboratory manifestations of pSS included in the EULAR Sjögren’s syndrome disease activity index (ESSDAI) [26] at diagnosis and during follow-up. Regarding glandular involvement, presence of xerostomia and xeropthalmia (graded on a VAS scale from 0 to 10), functional tests, including Schirmer’s test, Ocular Staining Score and Unstimulated Salivary Flow Rate (USFR), minor salivary gland biopsy (MSGB) results, including number of foci, focus score (FS) and GC-like structures’ presence, and PROs including ESSPRI were recorded.

Presence of extra-glandular organ involvement was defined according to ESSDAI definitions [26].

### 2.2. ILD Definition and Characterization

Presence of ILD was defined based on clinical, pulmonary function tests (PFTs) and imaging findings and was confirmed by revision of chest HRCT scans by an expert thoracic radiologist in all cases. Multidisciplinary team discussion was conducted only in case of diagnostic doubts on ILD etiology.

The thoracic radiologist classified the ILD pattern on HRCT into usual interstitial pneumonia (UIP), non-specific interstitial pneumonia (NSIP), organizing pneumonia (OP), lymphocytic interstitial pneumonia (LIP) or unclassifiable (NC) according to American Thoracic Society and European Respiratory society definitions [8,27]. ILD extension and severity was quantified visually on HRCT scans using the Warrick score [28]. Data regarding pulmonary signs and symptoms of ILD, as well as PFT results, were collected.

Based on the temporal relationship between ILD and pSS diagnosis, we identified a group of patients with ILD diagnosis preceding or occurring concomitantly with pSS diagnosis (ILD-onset), a group of patients developing ILD during the follow-up after pSS diagnosis (ILD-incident) and a group of pSS patients who never developed ILD (non-ILD). ILD-onset patients were originally referred to our outpatient clinic for the suspicion of an autoimmune systemic disease underling their pulmonary picture and were later diagnosed with pSS.

We than compared the demographic, clinical, serological and glandular characteristics of ILD-onset and ILD-incident patients. The pulmonary picture in terms of chest HRCT and functional tests was also compared between the two groups.

Finally, we compared the baseline characteristics of ILD-incident patients with those of pSS patients who did not develop ILD during follow-up (non-ILD group), in order to identify baseline risk factors able to predict ILD development in pSS patients.

### 2.3. Statistical Analysis

Data were expressed as median (IQR) for continuous variables and as absolute frequencies and percentages for categorical variables. Gaussian distribution of variables was assessed using Kolmogorov–Smirnov one-sample test. Chi-Square test and Student’s t-test were performed for comparisons of categorical variables and continuous variables, respectively. Non-parametric tests, such as the Kruskal–Wallis and Mann–Whitney tests, were used for variables that were not normally distributed.

Univariate and multivariate logistic regression analysis were performed to identify baseline risk factors for the development of ILD in pSS patients.

SPSS version 29.0.2.0 was used to perform statistical analysis.

## 3. Results

### 3.1. Study Population and PSS Patients’ Characteristics Associated with ILD

Out of 615 Sjogren’s patients in follow-up between January 2019 and September 2023, 98 were excluded because autoantibody testing with immunoblot panels was not available. A further 30 patients were excluded because pulmonary imaging and/or functional tests were not available. Finally, 13 pSS-ILD patients were excluded because they presented overlap with a second connective tissue disease (namely scleroderma in three cases, SLE in 2 cases, rheumatoid arthritis in two cases, anti-synthetase syndrome in four cases, mixed connective tissue disease in one case and ANCA-MPO vasculitis in 1 case).

Eventually, we included 474 patients (M: F = 28: 446), all fulfilling the ACR/EULAR 2016 criteria for pSS. Median age was 62 (IQR 51–72) years, and the median follow-up 6 (IQR 2–11) yrs. All enrolled patients were tested for autoantibodies presence by immunoblotting panels and the distribution of anti-Ro60 and anti-Ro52 specificities was the following: 242 (51.1%) patients exhibited double anti-Ro60/52 positivity, 70 (14.8%) presented isolated anti-Ro52 and 28 (5.9%) isolated anti-Ro60 autoantibodies, while 134 (28.3%) patients were seronegative.

Forty three out of the 474 enrolled patients were classified as pSS-ILD (M:F = 6:37), accounting for an overall ILD prevalence of 9.1% in our cohort. The median follow-up of pSS-ILD patients was 7 (IQR 4–15) years.

We observed a significantly higher prevalence of ILD in pSS patients testing positive for anti-Ro52 autoantibodies compared to anti-Ro52 negative patients (11.5% vs. 4.4%, *p* = 0.011). Moreover, male pSS patients presented ILD more frequently, with a prevalence of 21.4%, compared to 8.3% in female patients (*p* = 0.032). Finally, pSS-ILD patients were significantly older at the time of diagnosis of pSS (median age of 54.5 vs. 51.8 years, *p* < 0.001) and presented Raynaud’s phenomenon more frequently (*p* = 0.009).

Finally, pSS-ILD patients were more likely to receive treatment with oral steroids (*p* < 0.001) and traditional immunosuppressants, including Mycophenolate, Azathioprine and Cyclophosphamide (*p* < 0.001), while there was no statistically significant difference in the administration of Hydroxychloroquine (*p* = 0.182) and Rituximab (0.410).

### 3.2. PSS-ILD Patients’ Characteristics

Median age at the moment of ILD diagnosis was 68 years (IQR 63–74). Six out of 43 (13.9%) pSS-ILD patients were current or past smokers, 35/43 (81.4%) presented dyspnea and 32/43 (74.4%) complained of chronic cough. Thirty-one (72%) patients presented fine inspiratory crackles.

Nineteen out of 43 (44.2%) patients presented ILD as the first manifestation of pSS and were included in the ILD-onset group, while 24/43 (55.8%) were diagnosed with ILD after pSS diagnosis and composed the ILD-incident group. In the latter, the median latency from pSS to ILD diagnosis was 9 (IQR 3–14.2) years.

Based on chest HRCT findings, ILD pattern was defined as NSIP in 17/43 (39.5%), UIP in 8/43 (18.6%), OP in 2/43 (4.7%), LIP in 6/43 (14%), NSIP + OP in 8 (18.6%) and NC in 2/43 (4.7%) cases. Of note, both median age at pSS diagnosis and median age at ILD diagnosis were significantly different across the different HRCT pattern groups (*p* = 0.039 and *p* = 0.048, respectively), with UIP patients being the oldest both at pSS diagnosis (median age 69.5 years; IQR 56–68.3 years) and at the time of ILD diagnosis (median age 67 years; IQR 63.1–74.2 years). On the contrary LIP patients were the youngest at the time of pSS diagnosis with a median age of 49.5 years (IQR 30.4–68.5 years). Moreover, median latency between pSS diagnosis and ILD diagnosis tended to differ between the various pattern groups (*p* = 0.08), with LIP patients experimenting the longest time interval between pSS and ILD diagnosis (median 14 years; IQR 7.2–25.3 years).

**Figure 1:**
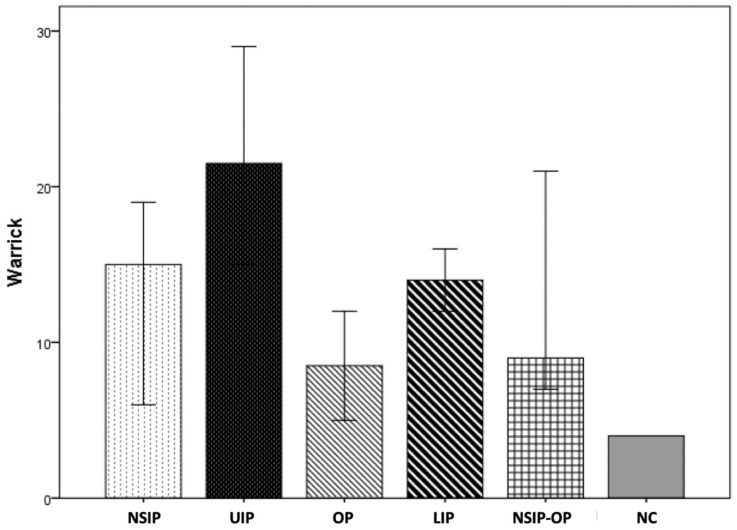
Graph showing the extent and severity of fibrosis according to Warrick score in pSS-ILD patients with different HRCT patterns. NSIP = interstitial pneumonia; UIP = usual interstitial pneumonia; OP = organizing pneumonia; LIP = lymphocytic interstitial pneumonia; NC = unclassifiable.

The extent of the pulmonary fibrosis was assessed visually on HRCT and the median Warrick score for the entire pSS-ILD group was 15.5 (IQR 7.5–19). Pulmonary function tests at baseline were only mildly compromised, with a median FVC of 85% (IQR 75–102%) and median baseline DLCO of 67% (IQR 59–77). The degree of pulmonary fibrosis on HRCT was not significantly different between pattern groups (*p* = 0.07), though UIP patients tended to present the highest Warrick score (median 21.5; IQR 15.9–27.4), as shown in Figure 1. In contrast, FVC and DLCO results were comparable across the different pattern groups (*p* = 0.692 and *p* = 0.543 respectively).

Regarding serology, double positivity of anti-Ro60/52 was the most frequent finding in pSS-ILD patients (60.5%), followed by isolated anti-Ro52 in 10/43 (23.2%). Interestingly 7/43 (16.3%) pSS-ILD patients were seronegative for anti-Ro60/52/La autoantibodies. However anti-nuclear autoantibodies were present in all seronegative subjects, as well as positive Rheumatoid Factor (RF) in two cases, anti-mitochondrial autoantibodies (AMA) in two patients, weak positivity of anti-PmScl 75 autoantibodies in two cases and anti-RNP autoantibodies in one case. In all pSS-ILD patients with atypical autoantibodies, overlapping Connective Tissue Diseases were thoroughly excluded, while 1 AMA positive patient was diagnosed with autoimmune biliary cholangitis. Finally, none of the pSS-ILD patients presented isolated anti-Ro60 autoantibodies.

Complete clinical and laboratory characteristics of pSS-ILD patients are presented in the Supplementary Table S1.

### 3.3. Comparison of ILD-Onset and ILD-Incident Phenotypes

When comparing the groups of ILD-onset and ILD-incident patients, the former tended to be more frequently of male sex (26.3% of cases vs. 4.2%, *p* = 0.07) and were significantly older at the time of pSS diagnosis (*p* = 0.002).

**Figure 2:**
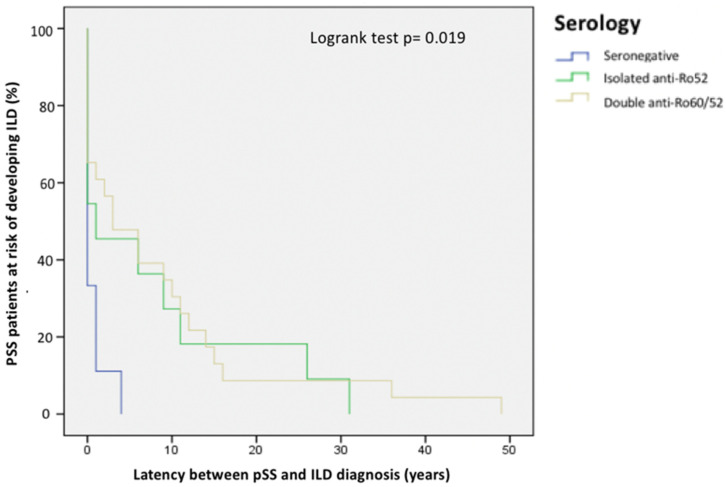
Kaplan-Meier curve showing the different latency from pSS diagnosis to ILD diagnosis between seronegative pSS ILD patients and anti-Ro52 + pSS ILD patients.

The anti-Ro/La autoantibodies profile did not significantly differ between the two groups (*p* = 0.262), though pSS-ILD patients with anti-Ro52+ autoantibodies (either isolated or combined with anti-Ro60 autoantibodies) were diagnosed with ILD after a significantly longer time since pSS diagnosis compared to seronegative pSS patients (*p* = 0.019) as shown in Figure 2.

Importantly, ILD-incident patients more often presented pSS-related hematologic abnormalities (*p* = 0.012) and cutaneous involvement (*p* = 0.027), inflammatory arthralgias (*p* = 0.026), C4 hypocomplementemia (*p* = 0.012) and positive RF (*p* = 0.031) compared to ILD-onset patients. Moreover, ILD-incident patients tended to more often present salivary gland enlargement (*p* = 0.07), purpura (*p* = 0.056) and hypergammaglobulinemia (*p* = 0.069), although these differences between the two groups were not statistically significant.

Regarding subjective sicca symptoms, ILD-onset patients reported significantly milder xeropthalmia and xerostomia on a 0–10 VAS scale (*p* = 0.020 and 0.050, respectively), while no significant difference was found in median ESSPRI (*p* = 0.918).

No other significant differences were observed among the two groups in terms of clinical and serologic characteristics, glandular functional tests and minor salivary glands Focus Score.

**Figure 3:**
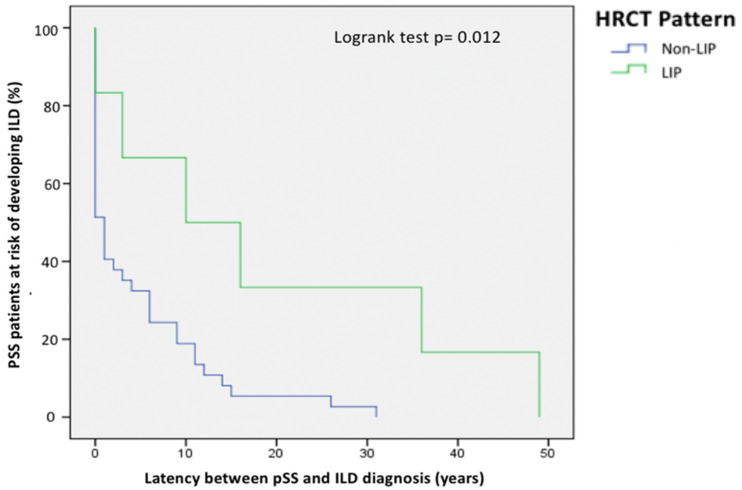
Kaplan-Meier curve showing the different latency from pSS diagnosis to ILD diagnosis between LIP patients and pSS-ILD patients with other HRCT patterns.

Regarding pulmonary involvement, the distribution of ILD patterns on HRCT was not significantly different between ILD-onset and ILD-incident patients, but 5 out of 6 LIP cases of our cohort occurred in the ILD-incident group and LIP patients were diagnosed with ILD after a significantly longer period of time since pSS diagnosis compared to ILD patients with other HRCT patterns, as shown in Figure 3.

Moreover, extension and severity of parenchymal fibrosis on HRCT were more pronounced in ILD-onset patients (*p* = 0.006).

Finally, ILD-incident patients were more likely to receive Hydroxychloroquine (*p* = 0.036), while there was no statistically significant difference in the administration of oral steroids (*p* = 0.420), traditional immunosuppressants (*p* = 0.267) and Rituximab (*p* = 0.129).

Complete clinical, laboratory and imaging data of ILD-onset and ILD-incident patients are reported in Table 1. 

### 3.4. Risk Factors for ILD Development

Out of 455 who did not present ILD at the time of pSS diagnosis, 24 developed ILD during follow-up (ILD-incident group). In order to identify clinical or serologic features potentially predicting the development of ILD in pSS, we compared the baseline characteristics of ILD-incident patients with those of pSS patients who did not develop ILD during follow-up (Non-ILD group).

Importantly, median follow-up time and prevalence of smoking history did not differ between ILD-incident and Non-ILD patients (*p* = 0.160 and *p* = 0.241).

The univariate logistic regression analysis showed that higher ESSDAI (*p* = 0.011), presence of Raynaud’s phenomenon (*p* = 0.009), anti-Ro52 autoantibodies (either isolated or combined with anti-Ro60, *p* = 0.031), hypergammaglobulinemia (*p* = 0.011), RF (*p* = 0.038) and C4 hypocomplementemia (*p* = 0.044) at baseline were associated with increased risk of ILD development during follow-up.

In the multivariate logistic regression analysis adjusted for smoking history and age at pSS diagnosis, ESSDAI (*p* = 0.05) and presence of Raynaud’s phenomenon (*p* = 0.013) at baseline were the only factors independently associated to ILD development.

Complete results of univariate and multivariate logistic regression analysis are shown in Table 2.

## 4. Discussion

In this retrospective observational study, 43 out of 474 pSS patients presented ILD, resulting in a pSS-ILD prevalence of 9.1%.

To the best of our knowledge, this is the largest monocentric pSS-ILD cohort to be described in Europe. The prevalence in our cohort is quite consistent with that reported by previous European studies, ranging from 5.7 to 13% [19,29,30] and considerably lower compared to reports from eastern Asia [20,21,31]. Such differences in prevalence may be influenced by ethnic factors, with Asian patients being more prone to develop ILD, as is the case for other CTDs such, as anti-MDA5+ dermatomyositis. However, selection biases, the definition of ILD and exclusion of cases of Sjogren’s syndrome overlapping with a second CTD may also play a role. For instance, studies based on registries of in-patient’s wards may select pSS patients with more severe clinical pictures and those more likely to present pulmonary involvement. Our pSS-ILD cohort comprised both patients originally diagnosed with ILD and referred to our outpatient clinic on suspicion of an underlying CTD and pSS patients followed in our outpatient clinic developing ILD during disease course. All ILD diagnoses were confirmed with revision of HRCT scans by an expert thoracic radiologist and cases of mild radiologic alterations without clinical relevance were excluded. Moreover, 13 pSS-ILD cases were excluded because of the presence of atypical autoantibodies and clinical manifestations consistent with the diagnosis of an overlapping CTD.

In keeping with previous reports, roughly half of the patients presented ILD as the first manifestation of pSS (ILD-onset) and the remaining cases were diagnosed with ILD after the diagnosis of pSS (ILD-incident) with a median latency from pSS to ILD diagnosis of 9 years [32,33].

When comparing the entire group of pSS-ILD patients to non-ILD pSS patients, the former were older at the time of pSS diagnosis, more frequently of male sex and they were more likely to present Raynaud’s phenomenon and positive anti-Ro52 autoantibodies. Male sex and older age at pSS diagnosis have been invariably associated both with ILD presence and poor outcome in pSS patients [19,22,29,34,35]. In contrast, the association of ILD with anti-SSA autoantibodies is less clear and seems to be related to anti-Ro52 specificity rather than anti-Ro60 [19,23], hence the importance of specifically assessing the presence of autoantibodies directed against the two autoantigens. Consistently with these findings, none of the 42 pSS-ILD patients in our cohort presented isolated anti-Ro60 autoantibodies.

Notably, however, in a recent metanalysis, older age at diagnosis, male sex and high C-reactive protein values were the only factors independently associated with ILD in pSS presence [15].

In this regard, we speculated that pSS-ILD encompasses distinct clinical phenotypes, which deserve to be further investigated. In more detail, there is preliminary evidence that ILD-onset patients represent a peculiar subset, with older age at diagnosis and a more severe respiratory picture at higher risk of progression [33,36]. Consistently, in our study, ILD-onset patients were older at the time of pSS diagnosis, tended to be more frequently male and presented more severe pulmonary fibrosis on HRCT when compared to ILD-incident patients. Of note, moreover, ILD-incident patients in this study more often presented more pSS-related hematologic, cutaneous and articular involvement, as well as reduced C4 serum levels and positive RF. They also tended to present a higher prevalence of salivary gland enlargement, purpura and hypergammaglobulinemia. It is noteworthy, that many of these clinical-serologic features are traditional prognostic factors in pSS and identify a phenotypic subset at higher risk of systemic involvement and lymphoma [37,38]. In 2018, Gao and colleagues performed a similar analysis comparing pSS-ILD patients with sicca-onset and pSS-ILD patients with non-sicca onset and found that the former more frequently had hypergammaglobulinemia and positive RF, as well as positive anti-SSA and -SSB [33].

Regarding pulmonary imaging, we found NSIP to be the most common ILD pattern on HRCT, occurring in 39% of patients. UIP pattern and a combination of NSIP and OP patterns were found in 18.6% of cases each, while LIP was the radiologic pattern in 14% of subjects. Finally, isolated OP patterns and NC pattern only accounted for 4.7% of the cases each. Indeed, NSIP is largely recognized to be the most common pSS-ILD pattern both on imaging and pathologic studies [39,40,41]. In contrast to a previous study which observed a higher prevalence of UIP in non-sicca onset pSS-ILD patients, we were not able to highlight a different distribution of HRCT pattern between ILD-onset and ILD-incident cases [33]. Interestingly, 5 out of 6 LIP cases of our cohort occurred in the ILD-incident group. In one exceptional case, LIP was recognized as the first manifestation of pSS in a 70-year-old woman, leading her to acute respiratory failure requiring admission to ICU. Moreover, in LIP patients, ILD diagnosis occurred after a mean latency of 19 years since pSS diagnosis, which is significantly longer compared to other HRCT patterns. Interestingly however, LIP patients usually present a long respiratory history characterized by acute episodes of cough and dyspnea with brilliant response to steroid therapy.

With respect to serologic findings, we observed double positivity of anti-Ro60/52 autoantibodies to be the most frequent in pSS-ILD cases. Isolated anti-Ro52 was found in 23% of patients, while 16% of subjects were seronegative for anti-Ro/La autoantibodies. The distribution of serologic specificities was comparable between ILD-onset and ILD-incident cases, but pSS-ILD patients with anti-Ro52+ autoantibodies exhibited a longer latency between pSS and ILD diagnosis compared to seronegative patients.

Finally, we focused on the 23 patients who developed ILD during the course of pSS follow-up and compared their clinical and serologic characteristics at the moment of pSS diagnosis with those of pSS patients who did not develop ILD, in order to identify potential predictive factors. Higher disease activity at baseline, presence of Raynaud’s phenomenon, anti-Ro52 autoantibodies (either isolated or combined with anti-Ro60), hypergammaglobulinemia, RF and C4 hypocomplementemia were identified as predictive factors for future ILD development. Higher disease activity and presence of Raynaud’s phenomenon at the moment of pSS diagnosis were the only independent risk factors identified by the multivariate analysis. These results suggest that these subsets of pSS patients should be carefully monitored and lung involvement should be periodically assessed during follow-up. Moreover, it could be speculated that early initiation of immunomodulatory therapy may play a role in preventing ILD development. However, further prospective studies would be needed to confirm this hypothesis.

To the best of our knowledge very few studies had specifically investigated baseline risk factors at the moment of pSS diagnosis for ILD-incident cases. Wang and colleagues performed a similar analysis on a very large cohort of Chinese pSS patients and found age, smoking and ANA-positivity to be independent risk factors for ILD development. However, in their study, patients were enrolled in the inpatient clinic, with a reported ILD prevalence as high as 78.6%. In contrast, our study addresses an Italian outpatient pSS cohort with a much lower ILD prevalence, which may explain the differences in the results.

The present study has some important limitations. First of all, data were collected retrospectively. Therefore, even if pSS-ILD patients were very well characterized, missing data may have affected the results. Particularly, an overlapping anti-synthetase syndrome may be challenging to diagnose in ILD dominant patients complaining of sicca symptoms.

In this regard, the complete Immunoblot panel for anti-sysnthetase autoantibodies was only available for 32 out of 43 enrolled patients. However, anti-Jo1 autoantibodies were tested in all enrolled patients by ELISA and, importantly, the complete Immunoblot panel was performed for all patients presenting clinical or radiological elements, raising the suspicion of an overlapping anti-synthetase syndrome (e.g., Raynaud’s phenomenon, arthritis, fever, OP or NSIP-OP pattern on HRCT, etc.).

Furthermore, defining pSS onset can be very challenging and pSS-ILD patients initially diagnosed with idiopathic ILD sometimes refer to a history of sicca symptoms when properly interrogated. However, for the purposes of this study we regarded such cases as ILD-onset because in our experience these patients report only mild sicca symptoms, have a clinical picture dominated by the respiratory involvement and therefore might represent a distinct subset of pSS-ILD [24]. Moreover, despite the fact that GERD is thought to contribute to pulmonary fibrosis development, complete data on GERD presence at baseline were not available for all non-ILD pSS patients. Therefore, we were not able to adjust the multivariable analysis for GERD presence when investigating predicting risk factors for ILD development.

On the other hand, this work presents several strengths. First of all, it enrolled the largest monocentric pSS-ILD cohort in Europe. PSS-ILD patients were well characterized and cases of Sjogren’s syndrome associated with a second CTD were thoroughly excluded. For this purpose, and in order to evaluate the relationship between anti-Ro sub specificities and ILD, we only included pSS patients for whom autoantibody testing with immunoblotting panels was available.

Finally, even if HRCT was not systematically performed for all pSS patients enrolled in the non-ILD group, in our outpatient clinic pSS patients in follow-up are periodically screened with Lung-Ultrasound, which has demonstrated a very high sensitivity for ILD detection. Therefore, the number of asymptomatic ILD cases potentially overlooked in the control group should be very limited.

## 5. Conclusions

ILD is relatively common in pSS with almost 1 out of 10 patients in our cohort presenting ILD in their disease course. PSS-ILD is heterogeneous both in clinical presentation and severity.

Proper phenotypic stratification of pSS-ILD patients is crucial both for research purposes and in clinical practice. ILD-onset and ILD-incident patients are likely to represent two distinct disease subsets exhibiting differences both in terms of pulmonary picture severity and extrapulmonary pSS-related organ involvement.

Presence of Raynaud’s phenomenon and elevated disease activity at pSS onset, especially in the biological domain, are risk factors for future ILD development, prompting careful follow-up in this subset of patients and intriguingly suggesting that immunomodulatory therapies may prevent ILD in these cases. Further studies are needed in order to test this hypothesis.

## Figures and Tables

**Table 1:** Comparison of clinical and laboratory features of ILD-*onset* and ILD-*incident* patients.

Patients Characteristics	ILD-Onset (n = 19)	ILD-Incident (n = 24)	*p*-Value
Age at pSS diagnosis, m (IQR)	70 (60.1–72.4)	57 (47.5–60.6)	**0.002**
Age at ILD diagnosis, m (IQR)	69 (58.4–71.3)	68 (61.5–70.2)	0.883
Sex male, n (%)	5/19 (26.3)	1/24 (4.2)	0.072
Smoking history, n (%)	3/19 (15.8)	3/24 (12.5)	1.000
GERD, n (%)	6/19 (31.6)	9/23 (39.1)	0.750
Constitutional symptoms, n (%)	0/19 (0)	2/24 (8.3)	0.495
Lymphadenopathy, n (%)	3/18 (16.7)	9/24 (37.5)	0.180
Salivary glands enlargement, n (%)	2/19 (10.5)	9/24 (37.5)	0.077
Articular involvement, n (%)	13/19 (15.8)	12/24 (50)	**0.026**
Cutaneous involvement, n (%)	0/19 (0)	6/24 (25)	**0.027**
Purpura, n (%)	0/19 (0)	5/24 (20.8)	0.056
Renal involvement, n (%)	0/19 (0)	2/24 (8.3)	0.495
Muscular involvement, n (%)	2/19 (10.5)	1/24 (4.2)	0.575
PNS involvement, n (%)	0/19 (0)	3/24 (12.5)	0.243
CNS involvement, n (%)	0/19 (0)	0/24 (0)	0.456
Hematologic involvement, n (%)	1/19 (5.3)	10/24 (41.7)	**0.012**
Non-Hodgkin Lymphoma, n (%)	1/19 (5.3)	2/24 (8.3)	1.000
Raynaud’s phenomenon, n (%)	11/19 (57.9)	12/24 (50)	0.760
Hypergammaglobulinemia, n (%)	7/19 (36.8)	16/24 (66.7)	0.069
C3 hypocomplementemia, n (%)	2/19 (10.5)	5/24 (20.8)	0.437
C4 hypocomplementemia, n (%)	0/19 (0)	7/24 (29.2)	**0.012**
Cryoglobulinemia, n (%)	0/19 (0)	4/23 (17.4)	0.118
ESSDAI, m (IQR)	10 (8.8–13.3)	9 (6.9–10.6	0.172
Double anti-Ro60/52, n (%)	8/19 (42.1)	15/24 (62.5)	0.262
Isolated anti-Ro52, n (%)	5/19 (26.3)	6/24 (25)	0.262
Isolated anti-Ro60, n (%)	0/19 (0)	0/24 (0)	0.262
Seronegative, n (%)	6/19 (31.6)	3/24 (12.5)	0.262
Anti-La, n (%)	6/19 (31.6)	11/24 (45.8)	0.369
RF, n (%)	5/19 (26.3)	15/24 (62.5)	0.031
MSGB FS, m (IQR)	1 (0.4–2)	1.2 (0.3–2.1)	0.642
FVC %, m (IQR)	79.4 (63.2–94)	94 (82.5–106.7)	0.207
DLCO %, m (IQR)	66 (51.2–79.4)	68.5 (62.9–76.1)	0.872
Warrick score, m (IQR)	18.5 (13.8–22-1)	11.5 (8–14)	**0.006**
NSIP, n (%)	7/19 (36.8)	10/24 (41.7)	0.337
UIP, n (%)	5/19 (26.3)	3/24 (12.5)	0.337
OP, n (%)	1/19 (5.3)	5/24 (20.8)	0.337
LIP, n (%)	1/19 (5.3)	5/24 (20.8)	0.337
NSIP + OP, n (%)	3/19 (15.8)	5/24 (20.8)	0.337
NC, n (%)	2/19 (10.5)	0/24 (0)	0.337

PNS = peripheral nervous system; CNS = central nervous system; NC = not classifiable; RF = rheumatoid factor; MSGB = minor salivary glands biopsy; FS = focus score; FVC = forced vital capacity; DLCO = diffusing capacity for carbon monoxide. Presence of SGE and extra-glandular pSS manifestations were defined according to ESSDAI definitions. Statistically significant *p*-values are marked in bold.

**Table 2:** Logistic regression analysis of baseline risk factors associated with ILD development.

Variables	Univariate Analysis		Multivariate Analysis	
	HR (95% CI)	*p*-Value	HR (95% CI)	*p*-Value
Age at pSS diagnosis	1.01 (0.98–1.05)	0.417		
Sex male	0.81 (0.10–6.26)	0.839		
Raynaud’s phenomenon	3.062 (1.323–7.07)	**0.009**	3.81 (1.83–10.88)	**0.013**
ESSDAI	1.12 (1.03–1.22)	**0.011**	1.10 (1–1.22)	**0.05**
Salivary glands enlargement	2.26 (0.96–5.33)	0.063		
Hypergammaglobulinemia	3.09 (1.29–7.34)	**0.011**		
RF	2.46 (1.05–5.75)	**0.038**		
C4 hypocomplementemia	2.58 (1.02–6.49)	**0.044**		
Anti-Ro52+	3.853 (1.13–13.12)	**0.031**		

RF = rheumatoid factor; anti-Ro52 positivity is intended as isolated or combined with anti-Ro60 (double positivity). Statistically significant *p*-values are marked in bold.

## Data Availability

Data are available upon reasonable request.

## Supplementary Materials

The following supporting information can be downloaded at: https://www.mdpi.com/article/10.3390/jcm13164963/s1, Table S1: Cumulative clinical and laboratory features of enrolled pSS-ILD patients.
