# Supplementary material for: Interstitial Lung Disease Phenotypes and Predictive Risk Factors in Primary Sjögren’s Syndrome
Source: J Clin Med. 2024 Aug 22;13(16):4963. doi: 10.3390/jcm13164963 (PMC11355583; doi:10.3390/jcm13164963)
Supplement: Supplementary file 1 [file jcm-13-04963-s001.zip › jcm-3149595-supplementary.pdf]

Supplementary Table S1 Cumulative clinical and laboratory features of enrolled pSS-ILD patients.

Patients characteristics	pSS-ILD patients
Age at pSS diagnosis, m (IQ)	64 (51–71)
Age at ILD diagnosis, m (IQ)	68 (63–74)
Sex male, n (%)	6/43 (14)
Smoking history, n (%)	6/43 (14)
Dyspnea, n (%)	35/43 (81.4)
Chronic cough, n (%)	32/43 (74.4)
Ocular dryness, n (%)	40/43 (93)
Oral dryness, n (%)	40/43 (93)
Constitutional symptoms, n (%)	2/43 (4.7)
Lymphadenopathy, n (%)	12/43 (27.9)
SGE, n (%)	11/43 (25.6)
Articular involvement, n (%)	11/23 (47.8)
Cutaneous involvement, n (%)	6/43 (14)
Renal involvement, n (%)	2/43 (4.7)
Muscular involvement, n (%)	3/43 (7)
PNS involvement, n (%)	3/43 (7)
CNS involvement, n (%)	0/43 (0)
Hematologic involvement, n (%)	11/43 (25.6)
Pulmonary involvement, n (%)	43/43 (100)
Raynaud's phenomenon, n (%)	17/43 (39.5%)
Non-Hodgkin Lymphoma, n (%)	3/43 (7)
Purpura	5/43 (11.6)
HRCT NSIP pattern, n (%)	17/43 (39.5)
HRCT UIP pattern, n (%)	8/43 (18.6)
HRCT LIP pattern, n (%)	6/43 (14)
HRCT NSIP-OP pattern, n (%)	8/43 (18.6)

HRCT OP pattern, n (%)	2/43 (4.7)
HRCT NC pattern, n (%)	2/43 (4.7)
MC, n (%)	5/43 (11.6)
C3 reduction, n (%)	7/43 (16.3)
C4 reduction, n (%)	7/43 (16.3)
Hypergammaglobulinemia, n (%)	23/43 (53.5)
Double Anti-Ro60, n (%)	23/43 (53.5)
Isolated anti-Ro52, n (%)	11/43 (25.6)
Isolated anti-Ro60, n (%)	0/43 (0)
Negative anti-Ro60/52, n (%)	9/43 (20.9)
Anti-La, n (%)	17/43 (39.5)
RF, n (%)	20/23 (46.5)
Cryoglobulinemia, n (%)	4/43 (9.3)
MSGB FS, m (IQ)	1.33 (1-2.6)

SGE= salivary glands enlargement; PNS= peripheral nervous system; CNS= central nervous system; NC= not classifiable; IgG= immunoglobulins class G; MC= monoclonal component; RF= rheumatoid factor; MSGB= minor salivary glands biopsy; FS= focus score.
Presence of SGE and extra-glandular pSS manifestations was defined according to ESSDAI definitions.
